# Supplementary material for: Copeptin Release in Arterial Hypotension and Its Association with Severity of Disease in Critically Ill Children
Source: Children (Basel). 2022 May 28;9(6):794. doi: 10.3390/children9060794 (PMC9222164; doi:10.3390/children9060794)
Supplement: Supplementary file 1 [file children-09-00794-s001.zip › children-1731496-supplementary.pdf]

Supporting Information S1

Individual characteristics of patients with systolic arterial hypotension with simultaneous low (<10 pmol/L) blood copeptin

Patient A: Male newborn (39 5/7 weeks of gestational age, Apgar score 4/6/7), 5 days old. Admission to PICU at 5th day of life following surgical correction of prenatally unknown interrupted aortic arch, aortic window, left pulmonary artery originating from aorta ascendens. Suspicion of CHARGE syndrome. Invasily ventilated for 264 h, non-invasive respiratory support for 147 h. Discharged from PICU on 19th day of stay. Developed hypotension (arterial blood pressure of 61/44/32 mmHg) at T4, while copeptin concentration was 6.1 pmol/L. No vasoactive support at that point of time.

Patient B: Female patient, 2 11/12 years old. Admission to PICU following removal of newly diagnosed medulloblastoma. Postoperatively invasively ventilated for 1.5 h; afterward, no need for non-invasive respiratory support. Discharged from PICU on 2nd day of stay. Developed hypotension (arterial blood pressure of 72/61/49 mmHg) at T1, while blood copeptin concentration was 4.2 pmol/L. No vasoactive support at that point of time.

Patient C: Male newborn (41 0/7 weeks of gestational age, Agar 8/9/9), 2 days old. Admission to PICU following surgical correction of total anomalous pulmonary venous drainage and ligation of persistent ductus arteriosus, followed by secondary thoraxic closure on first postoperative day. Postoperatively invasively ventilated for 118 h, non-invasive respiratory support for 4.6 h. Discharged from PICU on 10th day of stay. Developed hypotension (arterial blood pressure of 76/57/49 mmHg) at T5, while blood copeptin concentration was 8.3 pmol/L. No vasoactive support at that point of time.

Patient D: Male patient, 13 11/12 years old. Admission to PICU following surgical resection of a newly diagnosed meningioma. No respiratory support. Developed hypotension (systolic arterial blood pressure of 88/64/47 mmHg) at T1, while blood copeptin concentration was 9.4 pmol/L without vasoactive support.

Patient E: Male patient, 6 months old. Admission to PICU from emergency departement for increasing in- and expiratory stridor and oxygen requirement due to right descending aorta. After aortopexy, he was postoperatively invasively ventilated for 103 hours, no non-invasive respiratory support was necessary. Discharged from PICU on 15th day of stay. The patient was included before surgical correction; T0 took place in the morning before surgery. At T0, the patient developed hypotension (arterial blood pressure of 73/53/39 mmHg), while blood copeptin concentration was 8.7 pmol/L. No vasoactive support at that point of time.

Patient F: Male patient 7 11/12 years old. Admission to PICU following epipharyngeal bleeding during tonsillectomy. The patient was invasively ventilated for 25 h; subsequently, no need for non-invasive respiratory support. He was discharged on first postoperative day. He developed hypotension (arterial blood pressure of 76/55/36 mmHg) at T1, while blood copeptin concentration was 2.5 pmol/L. No vasoactive support at that point of time.

Patient G: Female patient, 4 months old, with dilatative cardiomyopathy. Admission to PICU following diagnostic cardiac catheterization. The patient did not need respiratory support and was discharged on first postinterventional day. She developed hypotension (arterial blood pressure of 71/48/38 mmHg) at T1, while blood copeptin concentration was 5.6 pmol/L. No vasoactive support at that point of time.

Patient H: Male patient, 7 months old, with exstrophy–epispadias complex, admitted to the PICU following second turn-in operation (first surgery was followed by a dehiscent abdominal suture) and epispadia correction. The patient was mechanically ventilated for 59 hours; he was discharged on 4th day after surgery. He developed hypotension (arterial blood pressure of 72/50/37 mmHg) at T3, while blood copeptin concentration was 6.8 pmol/L. He was under permanent infusions of noradrenaline at a rate of 0.05 µg/kg/min, morphine at a rate of 10 µg/kg/h and propofol at a rate of 3.75 mg/kg/h. Tracheal tube was pulled 4 h after T3 blood sample, and he was discharged from ICU 24 h later.

Table S1. Normal values for systolic blood pressure.

**Age Group**	**Systolic Blood Pressure mmHG**
0 days to 1 wk.	<59
1 wk. to 1 mo.	<79
1 mo. to 1 yr.	<75
2–5 yrs.	<74
6–12 yrs.	<83
13 to <18 yrs.	<90

Age-specific normal values (5th percentile) for systolic blood pressure [1,2].

References

1. Goldstein, B.; Giroir, B.; Randolph, A.; Members of the International Consensus Conference on Pediatric Sepsis. International pediatric sepsis consensus conference: Definitions for sepsis and organ dysfunction in pediatrics*. *Pediatr. Crit. Care Med.* **2005**, *6*, 2–8, doi:10.1097/01.pcc.0000149131.72248.e6.
2. Goldstein, B.; Giroir, B.; Randolph, A. Reply: Values for Systolic Blood Pressure. *Pediatr. Crit. Care Med.* **2005**, *6*, 500–501.
